# Apixaban versus other anticoagulants in patients with nonvalvular fibrillation: a comparison of all-cause and event-related costs in real-life setting in France

**DOI:** 10.1007/s10198-022-01513-2

**Published:** 2022-08-28

**Authors:** Manon Belhassen, Olivier Hanon, Philippe Gabriel Steg, Isabelle Mahé, Mélanie Née, Flore Jacoud, Faustine Dalon, François-Emery Cotté, Dominique Guitard-Dehoux, Claire Marant-Micallef, Eric Van Ganse, Nicolas Danchin

**Affiliations:** 1 PELyon, Lyon, France; 2 Service de Gériatrie, Université de Paris, APHP Centre, Hôpital Broca, 4468 Paris, EA France; 3 FACT, Université de Paris, INSERM U-1148/LVTS, F ; Assistance Publique-Hôpitaux de Paris, Hôpital Bichat, 75018 ParisParis, France; 4 APHP, Service de Médecine Interne, INNOVTE-FCRIN, Hôpital Louis Mourier, Université de Paris, Innovative Therapies in Haemostasis, INSERM, ColombesParisSaint Etienne, France; 5 Bristol-Myers Squibb, Rueil-Malmaison, France; 6 Département de Cardiologie, Hôpital Européen Georges Pompidou, Université de Paris, Paris, France

**Keywords:** Apixaban, Non-valvular atrial fibrillation, Costs, Anticoagulants

## Abstract

**Objectives:**

Compare costs associated with all-cause healthcare resource use (HCRU), stroke/systemic thromboembolism (STE) and major bleedings (MB) between patients with non-valvular atrial fibrillation (NVAF) initiating apixaban or other oral anticoagulants (OACs).

**Methods:**

We performed a retrospective cohort study using the French healthcare claims database, including NVAF patients between 2014/01/01 and 2016/12/31, followed until 2016/12/31. We used 4 sub-cohorts of OAC-naive patients, respectively initiating apixaban, dabigatran, rivaroxaban or VKAs. We matched patients initiating apixaban with patients initiating each other OACs using 1:n propensity score matching. All-cause HCRU and event-related costs by OAC treatment were estimated and compared between matched patients using generalised-linear models with gamma-distribution and two-part models.

**Results:**

There were 175,766 patients in the apixaban–VKA, 181,809 in the apixaban–rivaroxaban, and 42,490 in the apixaban–dabigatran matched cohorts. Patients initiating apixaban had significantly lower HCRU costs than patients initiating VKA (€1,105 vs. €1,578, *p* < 0.0001), dabigatran (€993 vs. €1,140, *p* < 0.0001) and rivaroxaban (€1,013 vs. €1,088 *p* < 0.0001). They have had significantly lower costs related to stroke/STE and MB than patients initiating VKA (respectively, €183 vs. €449 and €147 vs. €413; p < 0.0001), rivaroxaban (respectively, €145 vs. €197 and €129 vs. €193; *p* < 0.0001), and lower costs related to stroke/STE than patients initiating dabigatran (€135 vs. €192, *p* < 0.02). Costs related to MB were not significantly different in patients initiating apixaban and those initiating dabigatran (€119 vs. €149, *p* = 0.07).

**Conclusions:**

HCRU and most event-related costs were lower in patients initiating apixaban compared to other OACs. Apixaban may be cost-saving compared to VKAs, and significantly cheaper than other DOACs, although cost differences are limited.

**Supplementary Information:**

The online version contains supplementary material available at 10.1007/s10198-022-01513-2.

## Introduction

Atrial fibrillation (AF) is the most common type of cardiac arrhythmic disorder. Due to progresses in diagnosis and treatment of cardiovascular diseases and increasing life expectancy, the number of patients living with AF is predicted to grow substantially by 2030. In Europe, the prevalence is around 2% of the general population, i.e. double of that of the last decade [1]. In France in 2018, 226,000 patients newly treated with oral anticoagulants for AF were identified [2]. Non valvular atrial fibrillation (NVAF) is the most common form of AF.

Clinically, NVAF is associated with a high risk of mortality, stroke and systemic thromboembolic events (STE), but also with a high economic burden, mainly driven by hospitalizations: in France in 2012, cardiovascular-related hospitalizations of NVAF patients were associated with almost 2 billion euros, i.e., 2.6% of total expenditure in French hospitals [3].

Direct oral anticoagulants (DOACs) are indicated for prevention of stroke and STE in patients with NVAF. Since 2008, they have represented an alternative to the historical standard treatment, i.e. vitamin-K antagonists (VKAs), as they are easier to manage, no coagulation monitoring is required and drug and food interactions are limited [4]. More importantly, randomized trials have demonstrated the superiority of DOACs safety and at least similar efficacy compared to VKAs [5, 6]. VKAs and DOACs are now the two reference medications in patients with NVAF [7].

In France, apixaban is indicated since 2012 in patients with NVAF associated with at least one risk factor. Under request of the French Health Authority Transparency Committee, the use of apixaban in real-world practice has been demonstrated to be associated with better effectiveness, better safety, and lower all-cause mortality compared to the use of VKAs, and with superior safety than rivaroxaban, similar safety to dabigatran, and similar effectiveness than rivaroxaban in patients initiating oral anticoagulant (OAC) therapy between 2014 and 2016 [8].

As NVAF is associated with high economic burden and healthcare resource use (HCRU), it is also of interest to assess and compare HCRU and associated costs across various OACs treatments. Previous US studies described and compared HCRU and associated costs related to the use of VKAs or DOACs [9–12]. In France, cost-effectiveness studies have shown cost-effectiveness of DOACs[13] based on pivotal trials [6, 14]. However, there is no recent real-world study describing and comparing HCRU and costs associated to the use of the various types of OACs within the French health system.

This study aimed to compare HCRU and related costs associated to apixaban vs. VKAs, rivaroxaban or dabigatran in NVAF in France. More specifically, we described and compared the costs associated with all-cause HCRU, costs related to stroke/STE, and costs related to major bleedings between patients initiating apixaban vs. patients initiating VKA, rivaroxaban or dabigatran.

## Methods

### Study design and data sources

We conducted a retrospective cohort study from the French National Health System healthcare claims database (Système National des Données de Santé, SNDS), which contains anonymous individual information on patients’ sociodemographic characteristics, non-hospital reimbursed healthcare expenditures (e.g., drug dispensing, visits to physicians), and all hospital discharge summaries, including associated costs. Although no clinical, paraclinical, or biological data are recorded in the SNDS, the outcome events of interest for our study (STE and major bleedings) are available [15].

### Study population

The study population consisted of all patients aged ≥ 18 years diagnosed with NVAF, with at least one first reimbursement of OAC, i.e., VKAs, apixaban, rivaroxaban or dabigatran during the study period, i.e., between January 1, 2014 and December 31, 2016. The date of the first dispensing of OAC during this period was the index date. The selection of patients with NVAF and exclusion criteria have already been described before [8].

The study population consisted of 1:n propensity score matched patients initiating apixaban with patients initiating each other OAC. The matching process was based on propensity score (PS) matching described in detail in the previous paper [8]. Selected patients were followed during their exposure to the studied OAC treatment from the index date until end of follow-up, which was defined as: switch to another OAC treatment, treatment discontinuation, last patients’ health record (i.e., last care recorded in the database prior a 6-month period without any reimbursed care), date of death or end of the study period, whichever occurred first.

### Study outcomes and variables

We described the costs related to HCRU by OAC treatment cohort in matched OAC-naïve patients initiating either apixaban or other OACs. This included: costs and number of outpatient visits by type of visit (all, general practitioners, office-based cardiologists, office-based specialists, hospital-based physicians, nurse visits), packages for outpatient pharmacy, outpatient biology acts, medical procedures (separately for liberal activity, external activity of public hospitals and for inpatient procedures), and costs and cumulative length of stay of inpatient hospitalizations. We described hospitalization-related information by type of hospitalization: hospitalizations in short stay institutions (medicine, surgery, obstetrics [MCO]), home-based hospitalizations [HAD]; and hospitalizations related to after care and rehabilitation [SSR].

We also described costs related to: (1) stroke/STE and major bleedings costs defined as the first stroke/STE hospitalization or major bleeding acute care costs plus the costs of all subsequent hospitalizations related to the same type of event occurring within 3 following months. We identified subsequent hospitalizations using main, related, and associated diagnoses of hospital stays (ICD-10 codes); (2) the first event costs (either stroke/STE or major bleeding, i.e., any NVAF event) occurring over the follow-up period and costs of subsequent hospitalizations related to the same event within the 3 following months; and (3) major bleeding events by localization of the first bleeding event (gastrointestinal, intracranial or other). We defined a multisite localization when more than one major bleeding was identified during the same stay at two different localizations.

We estimated all costs from the medical care perspective, i.e., the costs of primary care estimated at the price actually paid and hospital costs valued as close as possible to the cost of production of the stays. Official tariffs were used to value pharmaceutical products, medical fees (consultations, visits, and procedures), laboratory, paramedical care, medical devices, medical transportations, ambulatory care. In France, a Diagnosis-Related Groups (DRG)-based payment system is used since 2004/2005 for funding acute services in all hospitals, differing by type of hospitalization [16], i.e., all stays with the same related diagnosis are assigned an unique and specific cost, whatever the actual costs are. Hospital admissions costs were obtained from the national cost studies (ENCC) carried out annually, which provide an average cost per DRG separately for public and private hospital [17].

### Statistical analyses

For each OAC treatment, we estimated the number and percentage of patients with: i) at least one reimbursement of a care, ii) at least one stroke/STE event during the follow-up, and iii) at least one major bleeding. For HCRU, we estimated the mean, standard deviation, median, percentiles 10 and 90 of the occurrence of each HCRU and associated costs in euros per patient per month (PPPM) in all patients, by OAC treatment. For costs related to stroke/STE and major bleeding events, we estimated the mean, standard deviation, median, percentiles 10 and 90 of costs in euros PPPM.

We compared HCRU and event-related costs between patients initiating apixaban matched to patients initiating other OACs using generalized linear models (G LM) with gamma distribution and log-link function. As gamma models are defined for positive values only, two-part models were used for items with no strictly positive costs and to account for the non-negligible part of the patient population without any event: the first part modeled the occurrence of the cost/event using a logistic regression, and the second part modeled the costs related to each HCRU/event, conditional on the HCRU/event occurrence, using a GLM with gamma distribution and log-link function. For all types of costs, we used the modified Park test to confirm that the data followed the gamma distribution.

All statistical analyses were performed using SAS (SAS Institute, North Carolina, US), version 9.4.

## Results

### Study population

There were 175,766 patients in the apixaban (*n* = 68,208)–VKA (*n* = 107,558) matched cohort, 181,809 in the apixaban (*n* = 81,759)–rivaroxaban (*n* = 100,050) matched cohort, and 42,490 in the apixaban (*n* = 21,245)–dabigatran (*n* = 21,245) matched cohort (see Fig. 1 of the supplementary material). The weighted standardized differences were < 10% for all confounding factors in the matched cohorts [8]. In all the three matched cohorts, the median follow-up time ranged from 186 to 220 days: 211 days (Q1: 72, Q3: 426) and 220 (Q1: 86, Q3: 481) days respectively, in the apixaban–VKA cohort, 214 days (Q1: 74, Q3: 439) and 205 (Q1: 60, Q3: 510) days respectively, in the apixaban–rivaroxaban cohort, and 213 days (Q1: 73, Q3: 436) and 186 (Q1: 61, Q3: 555) days respectively, in the apixaban–dabigatran cohort.

**Fig. 1:**
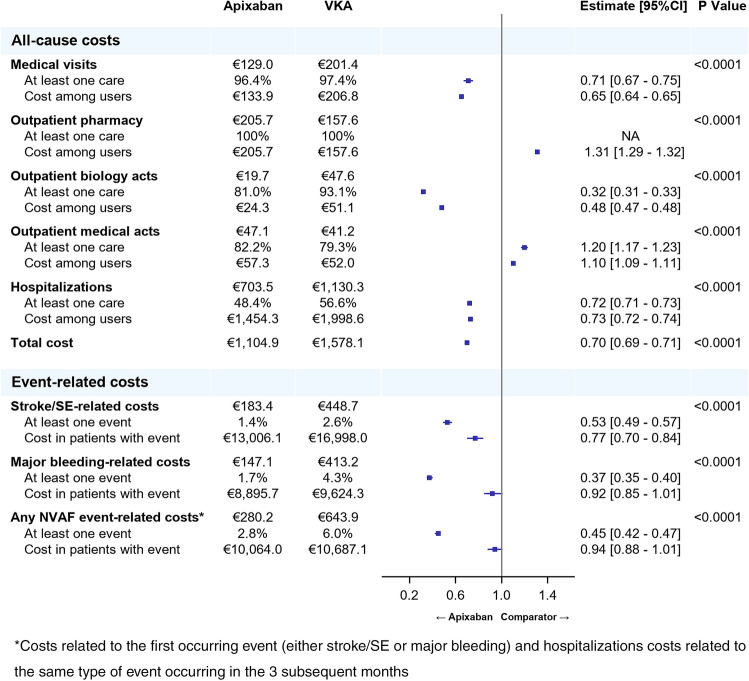
Comparison of all-cause HCRU-associated costs (pppm) and event-related costs over the follow-up period between apixaban and VKA matched cohorts (Generalized linear model with gamma distribution)

### Costs associated to all-cause healthcare resource utilization

Table 1 of supplementary material details mean HCRU during the follow-up period for apixaban and other matched OAC-Naive cohorts PPPM. Table 2 of supplementary material shows mean costs PPPM associated to all-cause HCRU during the follow-up period for apixaban and other OAC matched cohorts.

**Fig. 2:**
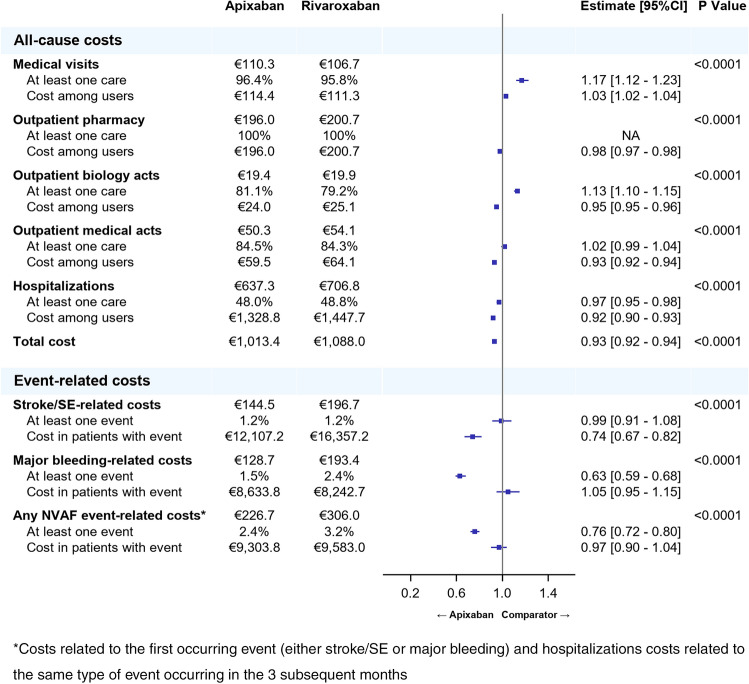
Comparison of all-cause HCRU-associated costs (PPPM) and event-related costs over the follow-up period between apixaban and rivaroxaban matched cohorts (Generalized linear model with gamma distribution)

**Fig. 3:**
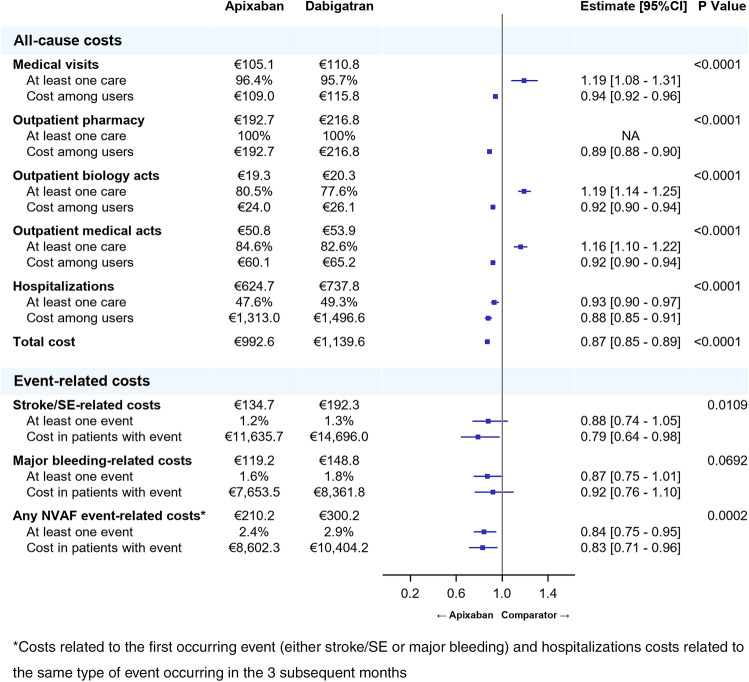
Comparison of all-cause HCRU-associated costs (PPPM) and event-related costs over the follow-up period between apixaban and dabigatran matched cohorts (Generalized linear model with gamma distribution)

Almost all the patients of each cohort were likely to incur costs related to medical visits (95.7% to 97.4%), a vast majority to biology acts (77.6% to 93.1%) and to medical acts (87.6% to 89.5%), and around half of them (47.6% to 56.6%) had costs related to hospitalizations at least once over the follow-up period (Figs. 1, 2, 3). Overall, the HCRU-associated costs of patients initiating apixaban were significantly lower than HCRU-associated costs of patients initiating VKAs (€1,105 vs. €1,578, *p* < 0.0001). The gap in HCRU-related costs between apixaban and VKAs was mainly driven by hospitalization costs (€704 vs. €1,130, respectively), and by nurse visits (€78 vs. €147, respectively, see supplementary material Table 2). However, costs related to outpatient pharmacy (€206 vs. €158, *p* < 0.0001) and medical acts (€47 vs. €41, *p* < 0.0001) were significantly higher in patients initiating apixaban than in patients initiating VKAs (Fig. 1). Similarly, HCRU-associated costs of patients initiating apixaban were significantly lower than those of patients initiating rivaroxaban (€1,013 vs. €1,088, *p* < 0.0001). Though differences were small, costs were significantly lower in patients initiating apixaban than in patients initiating rivaroxaban for all types of HCRU, except for medical visits, for which costs were higher in patients initiating apixaban (€110 vs. €107, *p* < 0.0001) (Fig. 2). HCRU-associated costs were also significantly lower in patients initiating apixaban compared to patients initiating dabigatran (€993 vs. €1,140, *p* < 0.0001) overall, as well as for costs related to each type of HCRU (Fig. 3).

### Event-related costs

Costs related to stroke/STE and major bleedings in patients initiating apixaban (respectively, €183 and €147) were significantly lower than in patients initiating VKAs (respectively, €449 and €413) (*p* < 0.0001 for both). Moreover, the first part of the model highlights the gap in the proportion of patients with at least one stroke/STE (1.4% in patients initiating apixaban vs. 2.6% in patients initiating VKAs) and one major bleeding (1.7% in patients initiating apixaban vs. 4.3% in patients initiating VKAs). For both events, costs were also significantly lower in patients with at least one event in patients initiating apixaban compared to those initiating VKAs, although the difference was smaller. The proportion of patients with at least one NVAF event (either stroke/STE or major bleeding) were lower in patients initiating apixaban than in patients initiating VKAs (2.8% vs. 6.0%, *p* < 0.0001), but related costs were similar in patients with at least one event in both cohorts, resulting in a lower overall cost of any NVAF event in patients initiating apixaban compared to patients initiating VKAs (i.e., the lower overall cost resulted from a lower number of events, with a similar cost per event in patients initiating apixaban compared to those initiating VKAs) (Fig. 1).

**Fig. 4:**
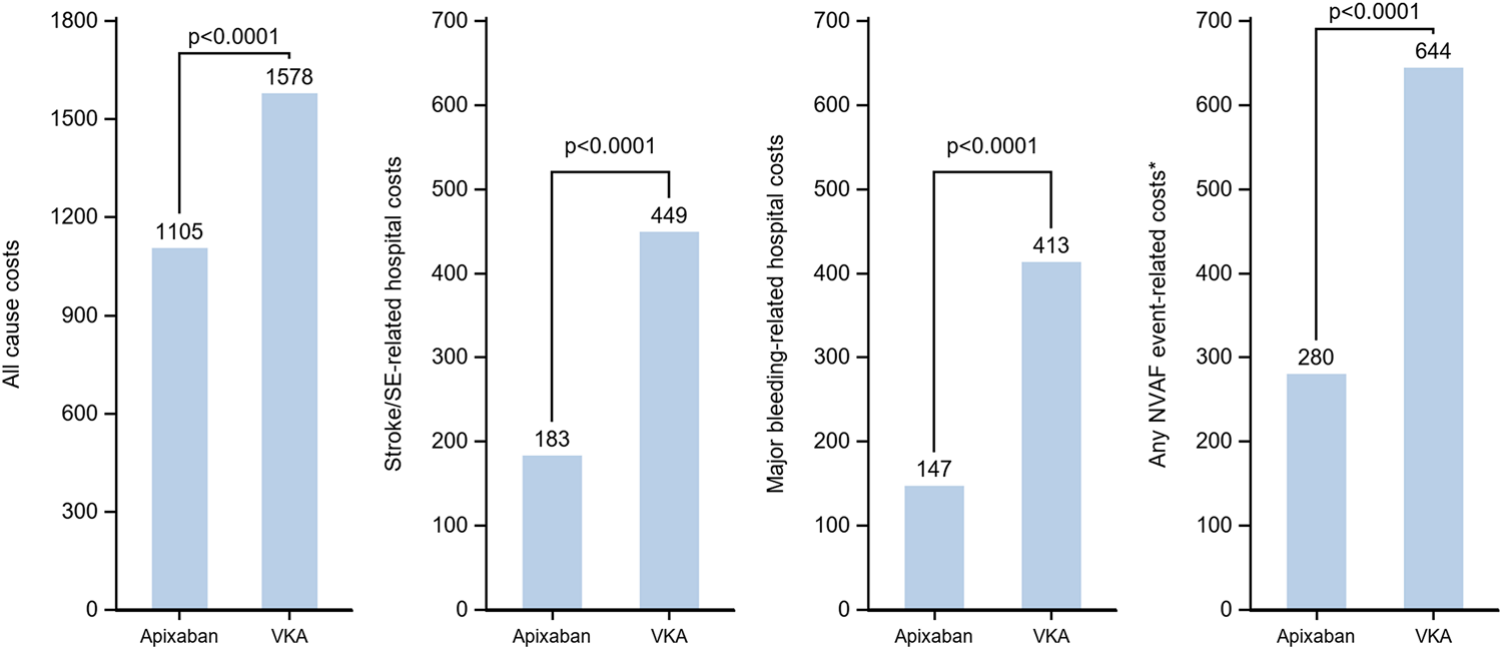
Comparison of costs (in €, PPPM) associated to all-cause HCRU, stroke/systemic embolism, major bleedings and any NVAF event over the follow-up period between apixaban and VKA matched cohorts

**Fig. 5:**
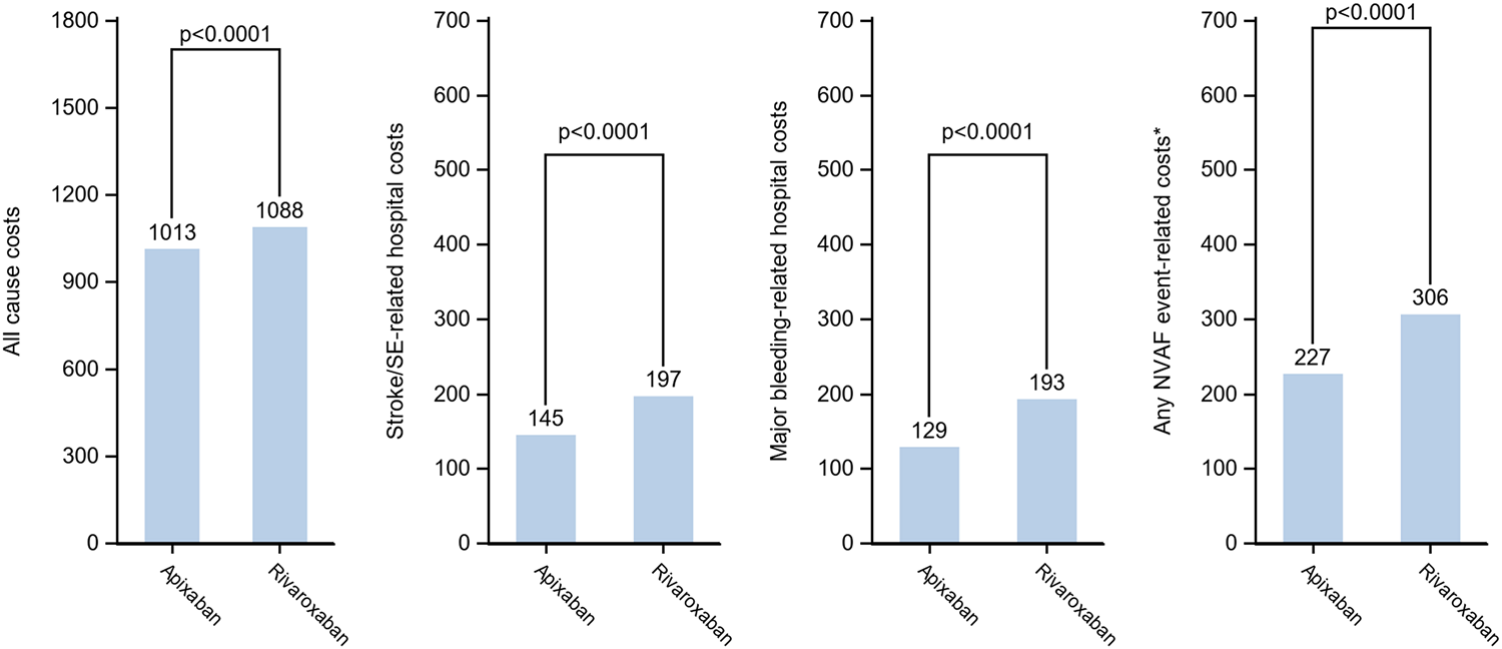
Comparison of costs (in €, PPPM) associated to all-cause HCRU, stroke/systemic embolism, major bleedings and any NVAF event over the follow-up period between apixaban and rivaroxaban matched cohorts

**Fig. 6:**
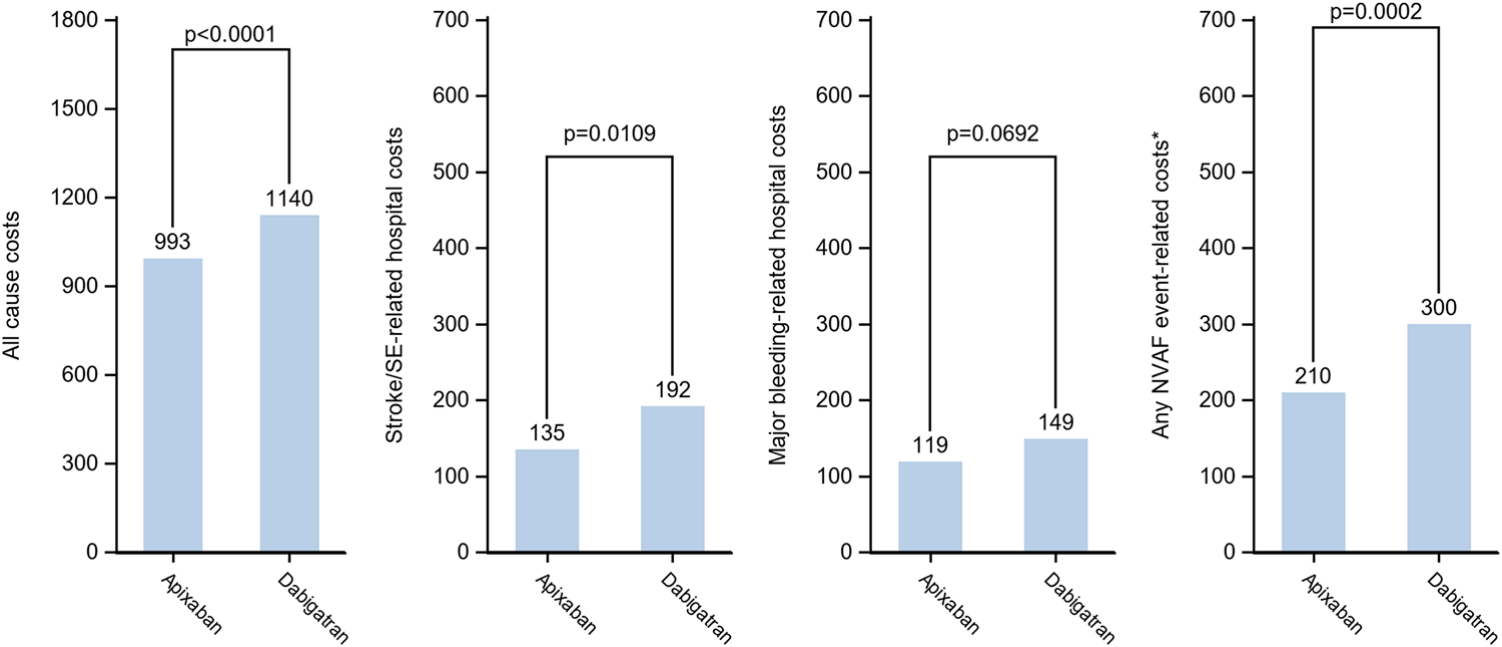
Comparison of costs (in €, PPPM) associated to all-cause HCRU, stroke/systemic embolism, major bleedings and any NVAF event over the follow-up period between apixaban and dabigatran matched cohorts

Costs related to stroke/STE and major bleedings in patients initiating apixaban (respectively, €145 and €129) were significantly lower than in patients initiating rivaroxaban (€197 and €193) (*p* < 0.0001 for both), despite the non-significant difference in the occurrence of stroke/STE between those two cohorts (1.2% of patients with at least one stroke/STE in both cohorts) (Fig. 2). However, the major bleeding-related costs in patients with one major bleeding was slightly higher in patients initiating apixaban than in patients initiating rivaroxaban (€8,634 vs. €8,243; IC = 0.95–1.15), but this difference was not statistically significant. As in the comparison to VKAs, the proportion of patients with at least one NVAF event (either stroke/STE or major bleeding) was lower in patients initiating apixaban than in patients initiating rivaroxaban (2.4% vs. 3.2%, *p* < 0.0001), but related costs were similar in patients with at least one event in both cohorts (Figs. 4, 5, 6).

Finally, costs related to stroke/STE in patients initiating apixaban (€135) were significantly lower than in patients initiating dabigatran (€192) (*p* = 0.0109), whereas the proportion of patients with the occurrence of at least one stroke/STE was not significantly different (respectively, 1.2% and 1.3% of patients). The proportion of patients with at least one major bleeding was similar in patients initiating apixaban (1.6%) and in patients initiating dabigatran (1.8%), and major bleeding-related costs were also not significantly different (respectively, €119 and €149, *p* = 0.0692). However, both the proportion of patients with any NVAF event (either stroke/STE or major bleeding) and related costs were significantly lower in patients initiating apixaban (2.4% of patients, €8,602) than in patients initiating dabigatran (2.9%, *p* < 0.0047, €10,404, *p* = 0.0122).

## Discussion

This is the first real-life study describing and comparing HCRU associated costs of apixaban vs. VKAs, rivaroxaban and dabigatran in France. Our results suggest that apixaban may be cost saving compared to all therapeutic alternatives. Overall, the proportion of users of medical resources was rather similar across DOACs, and lower in patients initiating DOACs than in those initiating VKAs. HCRU-related costs were lower in patients initiating apixaban than in patients initiating all other OACs. This finding was confirmed for costs related to stroke/STE which were lower in patients initiating apixaban than in patients initiating all other OACs, and as well, costs related to major bleeding were lower in patients initiating apixaban except in comparison to those initiating dabigatran. The difference in costs related to events in patients initiating apixaban compared to patients initiating other DOACs approximately corresponds to the cost of 1 month of DOAC treatment (~ €50). This is in line with the previously published comparable safety results [8]. Furthermore, the lower proportion of patients (and corresponding costs) with rehabilitation stays within patients initiating apixaban compared to other OACs strongly suggests a lower severity of NVAF-related events when patients initiate apixaban than other OACs [18]. Finally, it is remarkable that in patients with stroke/STE events, costs were consistently lower in patients initiating apixaban compared with all other OACs, possibly suggesting less severe events. On the contrary, the costs of bleeding events were similar whatever the OAC, suggesting comparable severity of bleeding events requiring hospitalization, irrespective of the OAC initially used.

To our knowledge, there is no other study describing and comparing costs of various OAC treatments in NVAF in France. However, overall, our results are in line with real-world studies in other countries comparing apixaban to VKAs or other DOACs [9–11]. Results may slightly differ because of differences in patient populations: indeed, some US studies were performed in elderly, or not fully representative populations due to the specific nature of US claims databases. For instance, in elderly populations, apixaban was associated with significantly lower hospitalization-related costs compared with other OACs (VKAs or other DOACs). Apixaban showed significantly lower all-cause HCRU costs and major bleeding-related medical costs [19]. In another study performed in the US Department of Defense population, overall, all-cause HCRU costs and event-related costs were significantly lower in patients initiating apixaban than in patients initiating rivaroxaban; and event-related costs were significantly lower in patients initiating apixaban than in those initiating VKAs or dabigatran [11]. Finally, Gilligan et al. showed lower outpatient and pharmacy-related costs and similar hospitalization costs in patients initiating apixaban compared to patients initiating dabigatran. Hospitalization costs were similar between the two cohorts in the US study, whereas they were lower in patients initiating apixaban in our study [10].

As the first real-world study describing and comparing HCRU and costs across various OACs used in NVAF, our study adds substantial information on costs related to OAC treatments in that condition in France, from a collective perspective. As an observational study, we described observed costs of the healthcare consumption occurrence actual year without using an index year for valued costs. Costs were observed during the period 2014–2016, during which DOACs costs have decreased by around 20%, while general health costs have increased of almost 20%. Consequently, our results would have been stronger if we would have indexed all costs on a specific year. As this study was requested by French health authorities, the associated protocol as well as the data collection and extraction have been validated by public institutions, ensuring the excellent quality of the data used [20]. Results are based on a large claims database, ensuring very high representativeness (> 98% of the population covered) and including comprehensive information on treatments and use of reimbursed healthcare resources. In addition, as the French covering system is universal, the data covers all types of populations, regardless of their age, social condition, or economic resources. Studies based on claims also provide large study populations with high statistical power and high-quality data not impacted by study conduct. Finally, contrary to other claims databases, the French claims database provides detailed information on different types of hospitalizations (distinguishing MCO, HAD, SSR) and types of physicians visited (public vs. private), allowing this study to provide crucial information to the payers on which type of costs are driving management costs in patients with NVAF.

However, the SNDS database only provides information on drug delivery, i.e., not on patients’ actual use, and no direct information about the daily prescribed dosages is available. The SNDS also does not contain data on drugs dispensed during hospital stays (except for very costly medication); however, the associated costs are considered in the hospitalization cost. Finally, as no diagnoses are associated to emergency visits, hospital-related costs may be underestimated, but this should be limited, as complications of NVAF often require in-patient hospitalizations.

As limited clinical information was available in the database, it was not possible to compare the distribution of risk factors between each cohort compared (e.g., smoking status, obesity, alcohol consumption, clearance of creatinine, chronic kidney disease). Matching using a large number of covariates in the PS improved control of confounding as these variables could collectively be proxies for non-collected factors.

Finally, as detailed in the previous paper [8], the following points may lead to selection biases: we used an algorithm and a proxy indicator to distinguish between non-valvular and valvular AF, which could lead to the selection of a more severe population. In addition, the description of comorbidities was performed using measures using proxies (i.e., CHADS2, CHADS2–VASc, modified version of HAS–BLED and Charlson scores), which could lead to misclassification of patients and underestimation of prevalence of these characteristics. However, both biases could not be avoided and were considered non-differential across studied cohorts.

## Conclusions

Together with our previous study comparing effectiveness and safety across the various anticoagulants used in NVAF, our study provides a comprehensive comparison (both clinical and economic) between apixaban vs. VKAs, rivaroxaban and dabigatran. In consistency to better effectiveness and safety and lower all-cause mortality, the initiation of apixaban in patients with NVAF was associated with lower costs compared to the initiation of VKAs, despite a wide difference in the drug price in favor of VKAs. Similarly, in addition to better safety and similar effectiveness than rivaroxaban, and similar safety to dabigatran, apixaban was associated with lower total HCRU-related costs and event-related costs for most of the events, although the magnitude of the difference in costs was much less than the difference observed with VKAs.

These findings suggest that apixaban may be cost-saving compared to all therapeutic alternatives in NVAF patients. This study provides crucial information about costs related to the management of NVAF patients treated with OACs.

## Supplementary Information

Below is the link to the electronic supplementary material.

Supplementary file1 (DOCX 60 KB)

## Data Availability

The database used cannot be shared. It is accessible only to authorized persons.
