# Supplementary material for: Apixaban versus other anticoagulants in patients with nonvalvular fibrillation: a comparison of all-cause and event-related costs in real-life setting in France
Source: Eur J Health Econ. 2022 Aug 28;24(6):867–75. doi: 10.1007/s10198-022-01513-2 (PMC10290596; doi:10.1007/s10198-022-01513-2)
Supplement: Supplementary file 1 — Supplementary file1 (DOCX 60 KB) [file 10198_2022_1513_MOESM1_ESM.docx]

**Supplementary material**

**Article title:**

Apixaban versus other anticoagulants in patients with nonvalvular fibrillation: a comparison of all-cause and event-related costs in real-life setting in France

**Journal name:** European Journal of Helath Economics

**Authors’ names:**

Manon Belhassen, PhD, Olivier Hanon, MD, Ph, Philippe Gabriel Steg, MD, PhD, Isabelle Mahé, MD, PhD, Mélanie Née, PhD, Flore Jacoud, MSc, Faustine Dalon, MA, François-Emery Cotté, PharmD, PhD, Dominique Guitard-Dehoux, PharmD, Claire Marant-Micallef, PharmD, Eric Van Ganse, MD, PhD, Nicolas Danchin, MD

**Corresponding author**

Manon Belhassen

210 avenue Jean Jaurès 69007 Lyon

[manon.belhassen@pelyon.fr](mailto:manon.belhassen@pelyon.fr)

Tel: 0033 4 81 09 96 07

**Content of the supplementary material:**

**Figure 1**. Population flowchart: population selection and matched cohorts

Table 1. All-cause healthcare resource utilization during the follow-up period for apixaban and other oral AC matched AC-Naive cohorts (PPPM)

Table 2. All-cause HCRU-associated costs during the follow-up period for apixaban and other oral AC matched AC-Naive cohorts (PPPM)


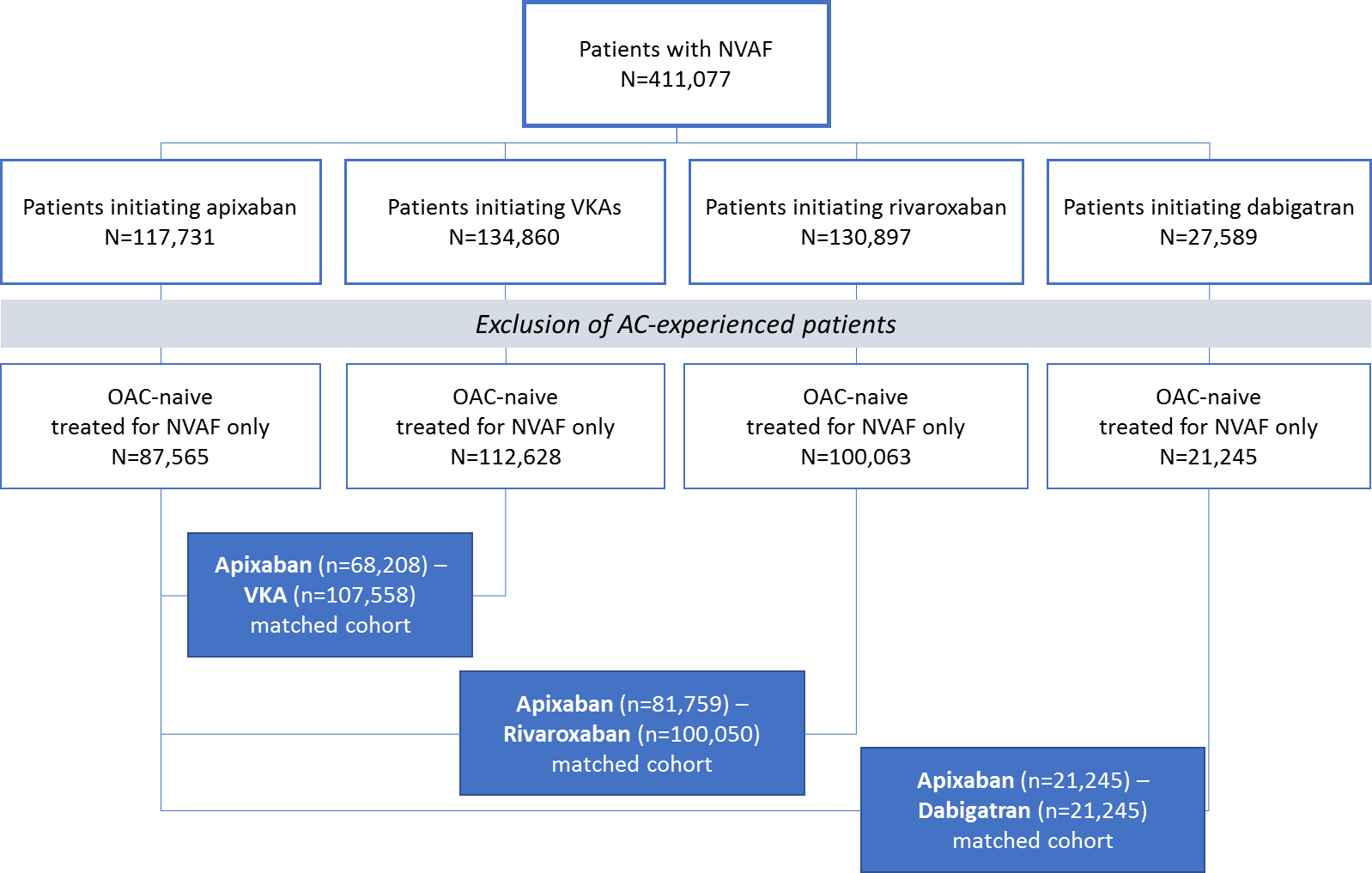


**Figure 1**. Population flowchart: population selection and matched cohort

*OAC: Oral anticoagulants*

**Table 1.** All-cause mean healthcare resource utilization during the follow-up period for apixaban and other OAC matched AC-Naive cohorts (PPPM)

|  | **Apixaban – VKAs** | | **Apixaban - Rivaroxaban** | | **Apixaban - Dabigatran** | |
| --- | --- | --- | --- | --- | --- | --- |
|  | **Apixaban (n=68,208)** | **VKAs (n=107,558)** | **Apixaban (n=81,759)** | **Rivaroxaban (n=100,050)** | **Apixaban (n=21,245)** | **Dabigatran (n=21,245)** |
|  | **Mean (SD)** | **Mean (SD)** | **Mean (SD)** | **Mean (SD)** | **Mean (SD)** | **Mean (SD)** |
| **Outpatient visits (any type)** | **5.1 (8.2)** | **8.5 (9.8)** | **4.4 (7.4)** | **4.2 (7.1)** | **4.2 (7.1)** | **4.3 (7.2)** |
| General practitioners | 1.1 (1.3) | 1.2 (1.4) | 1.0 (1.2) | 1.0 (1.3) | 1.0 (1.3) | 1.1 (1.5) |
| Office-based cardiologists | 0.2 (0.8) | 0.2 (0.7) | 0.3 (0.9) | 0.3 (1.0) | 0.3 (0.9) | 0.3 (1.0) |
| Other office-based specialists | 0.2 (0.5) | 0.2 (0.6) | 0.2 (0.5) | 0.2 (0.5) | 0.2 (0.4) | 0.2 (0.5) |
| Private hospital-based physicians | 0.0 (0.3) | 0.0 (0.4) | 0.0 (0.3) | 0.0 (0.3) | 0.0 (0.2) | 0.0 (0.4) |
| Public hospital-based physicians | 0.1 (0.4) | 0.2 (0.4) | 0.1 (0.4) | 0.1 (0.4) | 0.1 (0.4) | 0.1 (0.5) |
| Nurses | 3.5 (7.8) | 6.8 (9.4) | 2.7 (7.0) | 2.5 (6.6) | 2.5 (6.7) | 2.5 (6.7) |
| **Outpatient pharmacy (any type)** | **14.4 (19.5)** | **17.1 (26.3)** | **13.1 (17.6)** | **12.9 (17.9)** | **12.8 (18.4)** | **13.5 (20.5)** |
| OAC treatment | 1.4 (2.1) | 1.6 (3.0) | 1.4 (2.0) | 1.5 (2.5) | 1.4 (2.0) | 1.7 (4.5) |
| **Outpatient biology act (any type)** | **2.9 (4.9)** | **6.0 (6.7)** | **2.8 (5.0)** | **2.8 (5.6)** | **2.8 (4.9)** | **2.9 (6.4)** |
| Prothrombin time for VKAs treated patients | 0.0 (0.2) | 2.4 (1.7) | 0.0 (0.2) | 0.0 (0.2) | 0.0 (0.2) | 0.0 (0.2) |
| **Medical procedure (any type)** | **1.9 (4.4)** | **2.4 (9.4)** | **2.0 (13.6)** | **2.1 (8.4)** | **1.9 (4.3)** | **2.1 (6.6)** |
| External activity | 0.2 (0.6) | 0.2 (0.6) | 0.2 (0.6) | 0.2 (0.9) | 0.2 (0.5) | 0.2 (0.7) |
| Hospital activity | 1.0 (4.2) | 1.7 (9.2) | 1.0 (13.5) | 1.1 (8.2) | 1.0 (4.1) | 1.1 (6.2) |
| Liberal activity | 0.7 (1.1) | 0.6 (1.1) | 0.8 (1.2) | 0.8 (1.4) | 0.8 (1.2) | 0.8 (1.6) |
| **Hospitalizations (any type): number of stays** | **0.2 (0.5)** | **0.3 (1.1)** | **0.2 (0.5)** | **0.2 (0.6)** | **0.2 (0.5)** | **0.2 (0.6)** |
| HAD | 0.0 (0.0) | 0.0 (0.0) | 0.0 (0.0) | 0.0 (0.0) | 0.0 (0.0) | 0.0 (0.0) |
| MCO | 0.2 (0.5) | 0.3 (1.1) | 0.2 (0.5) | 0.2 (0.6) | 0.2 (0.5) | 0.2 (0.6) |
| SSR | 0.0 (0.0) | 0.0 (0.1) | 0.0 (0.0) | 0.0 (0.1) | 0.0 (0.0) | 0.0 (0.1) |
| **Hospitalizations (any type): length of stays** | **1.2 (4.3)** | **2.5 (9.2)** | **1.0 (4.4)** | **1.3 (13.8)** | **1.0 (3.7)** | **1.3 (7.2)** |
| HAD | 0.0 (0.9) | 0.1 (4.2) | 0.0 (0.8) | 0.0 (0.5) | 0.0 (0.1) | 0.0 (0.9) |
| MCO | 1.1 (3.4) | 2.0 (7.2) | 1.0 (3.7) | 1.1 (13.5) | 0.9 (3.4) | 1.1 (6.4) |
| SSR | 0.1 (2.2) | 0.4 (3.6) | 0.1 (2.0) | 0.2 (2.5) | 0.1 (1.1) | 0.2 (2.7) |

*Out-hospital; Number of drug packages

OAC: Oral Anticoagulant Treatment; HAD: HAD: home-based hospitalizations; MCO: hospitalizations in short stay institutions (medicine, surgery, obstetrics); SSR: hospitalizations related to after care and rehabilitation

Table 2. All-cause healthcare resource utilization associated mean costs during the follow-up period for apixaban and other OAC matched AC-Naive cohorts (PPPM)

|  | **Apixaban – VKAs** | | | **Apixaban - Rivaroxaban** | | **Apixaban - Dabigatran** | | |
| --- | --- | --- | --- | --- | --- | --- | --- | --- |
|  | **Apixaban (n=68,208)** | **VKAs (n=107,558)** | **Apixaban (n=81,759)** | | **Rivaroxaban (n=100,050)** | | **Apixaban (n=21,245)** | **Dabigatran (n=21,245)** |
|  | **Mean (SD)** | **Mean (SD)** | **Mean (SD)** | | **Mean (SD)** | | **Mean (SD)** | **Mean (SD)** |
| **Outpatient visit (any type)** | **129.0 (260.2)** | **201.4 (322.5)** | **110.3 (229.0)** | | **106.7 (213.8)** | | **105.1 (207.2)** | **110.8 (217.6)** |
| General practitioners | 29.7 (40.3) | 35.7 (43.9) | 27.5 (38.5) | | 27.5 (38.2) | | 27.3 (36.6) | 29.4 (44.3) |
| Office-based cardiologists | 8.9 (28.2) | 6.7 (26.5) | 10.7 (32.3) | | 12.1 (36.9) | | 10.8 (31.3) | 13.4 (39.8) |
| Other office-based specialists | 7.3 (19.0) | 6.8 (20.0) | 7.7 (19.1) | | 8.4 (21.3) | | 7.9 (20.0) | 7.7 (19.1) |
| Private hospital-based physicians | 0.7 (8.0) | 0.9 (9.3) | 0.7 (8.2) | | 0.7 (7.6) | | 0.5 (5.1) | 0.8 (10.3) |
| Public hospital-based physicians | 4.0 (11.0) | 4.6 (11.7) | 3.8 (11.0) | | 4.1 (13.9) | | 3.8 (11.2) | 4.0 (14.1) |
| Nurses | 78.3 (246.8) | 146.7 (310.0) | 59.9 (214.5) | | 53.8 (198.1) | | 54.7 (193.1) | 55.6 (199.8) |
| **Outpatient pharmacy (any type)** | **205.7 (379.9)** | **157.6 (339.4)** | **196.0 (350.6)** | | **200.7 (329.5)** | | **192.7 (328.3)** | **216.8 (422.7)** |
| AC treatment | 95.0 (122.1) | 10.8 (54.9) | 94.1 (120.4) | | 97.7 (150.4) | | 93.7 (122.2) | 110.0 (254.5) |
| **Outpatient biology acts (any type)** | **19.7 (32.2)** | **47.6 (46.7)** | **19.4 (33.7)** | | **19.9 (40.3)** | | **19.3 (33.9)** | **20.3 (43.8)** |
| Prothrombin time for VKAs treated patients | 0.1 (0.9) | 12.7 (9.1) | 0.1 (0.9) | | 0.1 (0.9) | | 0.1 (0.9) | 0.1 (0.9) |
| **Medical procedures (any type)** | **47.1 (102.5)** | **41.2 (104.3)** | **50.3 (103.7)** | | **54.1 (126.0)** | | **50.8 (100.6)** | **53.9 (135.8)** |
| External activity | 10.0 (31.2) | 10.1 (33.1) | 9.5 (32.1) | | 9.7 (42.0) | | 9.3 (31.6) | 9.4 (37.3) |
| Liberal activity | 37.1 (97.5) | 31.1 (98.2) | 40.9 (98.1) | | 44.4 (118.6) | | 41.5 (95.7) | 44.5 (130.6) |
| **Hospitalizations (any type)** | **703.5 (2,085.7)** | **1,130.3 (3,043.7)** | **637.3 (2,140.3)** | | **706.8 (4,437.7)** | | **624.7 (2,048.5)** | **737.8 (2,797.0)** |
| HAD | 1.7 (159.9) | 14.8 (859.4) | 1.2 (140.1) | | 2.3 (115.7) | | 0.6 (31.3) | 5.9 (236.3) |
| MCO | 682.3 (2,025.8) | 1,018.2 (2,729.9) | 619.9 (2,093.8) | | 664.8 (4,370.9) | | 608.5 (1,998.1) | 676.2 (2,664.0) |
| SSR | 19.5 (315.3) | 97.3 (795.9) | 16.2 (269.2) | | 39.7 (613.0) | | 15.6 (263.5) | 55.7 (628.0) |
| **Total HCRU-associated costs** | **1,104.9 (2,199.8)** | **1,578.1 (3,137.7)** | **1,013.4 (2,246.2)** | | **1,088.0 (4,511.0)** | | **992.6 (2,150.8)** | **1,139.6 (2,899.6)** |
| **Stroke/STE related costs**^*^ | 183.4 (5,522.5) | 448.7 (8,355.0) | 144.5 (3,174.1) | | 196.7 (5,305.9) | | 134.7 (3,335.3) | 192.3 (4,268.4) |
| **Major bleeding related costs** |  |  |  | |  | |  |  |
| Overall | 147.1 (3,510.6) | 413.2 (7,046.3) | 128.7 (3,296.3) | | 193.4 (5,051.0) | | 119.2 (2,110.5) | 148.8 (2,151.7) |
| Gastrointestinal | 29.3 (741.4) | 88.5 (3,770.3) | 25.4 (701.0) | | 54.6 (2,467.5) | | 31.4 (859.2) | 69.5 (1,464.8) |
| Intracranial | 80.7 (3,032.9) | 252.4 (5,829.8) | 72.5 (2,869.0) | | 90.3 (4,307.6) | | 64.2 (1,846.2) | 43.3 (1,347.6) |
| Other | 35.9 (1,603.3) | 69.1 (1,227.7) | 29.9 (1,462.1) | | 47.3 (926.5) | | 23.2 (557.4) | 34.0 (816.2) |
| Multisite | 1.2 (136.4) | 3.3 (189.6) | 1.0 (124.6) | | 1.1 (185.6) | | 0.5 (70.5) | 2.0 (127.4) |
| ^*^ Costs related to hospitalizations only  OAC: Anticoagulant Treatment; HAD: home-based hospitalizations; MCO: hospitalizations in short stay institutions (medicine, surgery, obstetrics); SSR: hospitalizations related to after care and rehabilitation; HCRU: Healthcare Resources Utilisation | | | | | | | | |
